# Reply to: Assessing the precision of morphogen gradients in neural tube development

**DOI:** 10.1038/s41467-024-45149-7

**Published:** 2024-02-01

**Authors:** Roman Vetter, Dagmar Iber

**Affiliations:** 1 Department of Biosystems Science and Engineering, ETH Zürich, Schanzenstrasse 44, 4056 Basel, Switzerland; 2 Swiss Institute of Bioinformatics, Schanzenstrasse 44, 4056 Basel, Switzerland

**Keywords:** Pattern formation, Computational biology and bioinformatics

**replying to** M. Zagorski et al. *Nature Communications* 10.1038/s41467-024-45148-8 (2024)

In a recent article^1^, we demonstrated that single morphogen gradients in the developing mouse neural tube (NT) can carry sufficient positional accuracy to explain the patterning precision of progenitor domain boundaries. Zagorski et al. had previously concluded otherwise^2^, based on methodological inconsistencies that we have revealed. The authors now comment on our work with a Matters Arising letter. We rebut their criticism point by point in the Supplement, and summarize the main aspects here.

The authors criticize our comparison of the different analysis methods (FitEPM, NumEPM, DEEM) using a set of exponential functions. It is a fundamental part of quantitative science to validate all methods used to process data—be it part of experimental, theoretical or numerical work—against known results. If a method fails to provide the correct results for known problems, it is unreasonable to apply it to similar problems, for which the answer is not known. The authors’ indirect approximation (FitEPM) leads to a vast overestimation of the positional error in case of exponential gradients, and is accurate only near the morphogen source^1^. The same limitations apply to noisy gradient data, and the challenges of background subtraction and smoothening apply to all three methods.

The authors insist that further away from the source the observed gradients are flat, such that our arguments would not apply. First, their approximation (FitEPM) will yield the wrong positional error whenever the mean deviates substantially from an exponential^1^ (i.e., also in flat parts of the gradient), as it makes use of the exponential shape explicitly. Second, the flat part of the gradients is not reliable biological data. We speculated in our paper that the switch to a flat gradient shape may be due to insufficient imaging depth, but the employed imaging depth remained unknown to us^1^. We have since received confirmation that Zagorski et al. indeed employed 8-bit imaging (A. Kicheva, personal communication; Supplementary Information). 8-bit imaging only permits the detection of a 2^8^ = 256-fold intensity change. As such, it is technically impossible to detect an exponential gradient beyond 5.5 times its decay length (~110 µm) from the source, which coincides with the point where Zagorski et al. find the transition from an exponential to a flat gradient shape. The physical limits of their imaging setup and the mathematical limitation of their approximation of the positional error make it impossible to evaluate the positional error of the gradients at later time points or further away from the source.

A key conclusion of Zagorki et al.^2^ is that morphogen gradients are too noisy to specify cell fate in the NT beyond the first 30 h. The authors now argue that their analysis must be considered correct because it 1) provides an explanation for the sensitivity of the progenitor markers to both SHH and BMP, and 2) is consistent with their previous postulate that cell differentiation rather than morphogen gradients define the progenitor domain boundary positions at later stages^3^. However, one can imagine other roles than precision for this parallel SHH/BMP input^4^, and their own work^3^ and that by others^5^ showed that the key marker of the motor neuron domain, OLIG2, remains sensitive to SHH signaling also at later stages. The limits posed by 8-bit imaging and the inaccuracy of the chosen error approximation are rigorous mathematical facts. They cannot be challenged biologically.

Given that the experimental data cannot be used beyond the 8-bit limit, we developed computational approaches to estimate the gradient variability further away from the source based on available experimental measurements^1^. This necessitated assumptions, which the authors now question. For one, based on error propagation, we determined the expected gradient variability beyond the 8-bit detection limit for the case that gradients maintain their measured exponential shape across the domain. Secondly, we developed a cell-based simulation framework that allows us to estimate the positional error from measured molecular noise levels. Both the error propagation method and the cell-based simulations showed that gradients remain sufficiently precise such that gradients can, in principle, pattern the NT throughout its development.

Zagorski et al. claim that it is unrealistic to assume that gradients remain exponential. However, they use exponential gradients, rather than their measured gradients, in their own paper^2^, both in their error propagation method (FitEPM), and also as input when evaluating the potential of opposing gradients in NT patterning via their decoding map, as the reported gradients are too flat and noisy outside the 8-bit limit, also when both gradients are considered simultaneously. As we showed in follow-up work, our results also apply to non-exponential gradients^6^, and gradient precision is substantially higher still when taking into consideration that morphogens spread at least in 2D, rather than 1D^7^.

The authors further claim that our statistical approach was flawed because the gradient amplitude and decay length of exponential gradients would be correlated. As the authors only provide a correlation coefficient but no data, it remains unclear what data they base their statement on. Consistent with data of the SHH gradient in the mouse NT^8^ that we based our analysis on (Pearson’s R = −0.0061, Kendall’s τ = 0.056^1^), our cell-based model shows that such a correlation, while expected in a deterministic setting, is negligibly small with independent cell-to-cell molecular noise (Fig. 1).

**Fig. 1:**
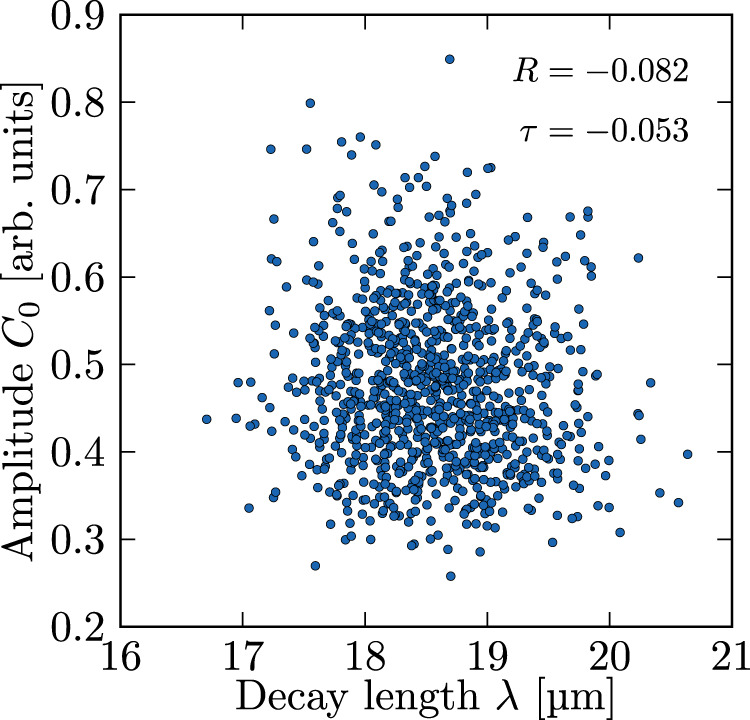
Absence of correlation between gradient amplitude and decay length in a cell-based model with independent cell-to-cell molecular noise. Data from *n* = 1000 simulated gradients obtained with independent noise between individual cells in all three kinetic parameters, CV_*p,d,D*_ = 0.3. See^1^ for method details. Pearson’s R = −0.082 (*p* = 0.010), Kendall’s τ = −0.053 (*p* = 0.012).

As we analyzed the processed gradient data that we received from the authors, we noticed that the gradients had been binned from five somite stages and scaled to the same domain length before determining the positional error. This introduces an artificial positional error, which can explain the remaining small deviation in the positional error of gradients and readouts. The authors claim that we overestimate this artificial positional error as the errors may not be fully additive. With the unprocessed data inaccessible to us, the degree of additivity of this artificial error in the authors’ methodology on the biological positional error remains unknown. However, as we show in our article, the positional error of the gradients and the readout are comparable even if the errors are only partially additive, as the difference is rather small.

Zagorski et al. criticize that we did not also correct the readout data. The method section of their Science paper^2^ mentions the scaling neither for the gradients nor for the readout, and the raw data is not published. We therefore measured the positional error of the dorsal NKX6.1 boundary ourselves. We could reproduce their results only if we bin, but do not scale the domains, from which we concluded that the authors’ readout dataset was likely not scaled. During the writing of our response to their Matters Arising letter, the authors informed us that they did not scale the readout data (A. Kicheva, personal communication; Supplementary Information). With the data and scripts that we have access to we cannot check this. While we remain interested in settling the point, we consider domain scaling a minor issue, given the considerable challenges in detecting gradients and aligning them with their readouts, in particular in pseudostratified epithelia, where nuclei are not perfectly aligned with their apical surface^9^.

In conclusion, none of the points raised by Zagorski et al. bear relevance to our conclusions. The remaining uncertainties, whilst in principle addressable, cannot be resolved with the data and method details that we have access to. See Supplementary Information for the detailed rebuttal.

## Reporting summary

Further information on research design is available in the Nature Portfolio Reporting Summary linked to this article.

### Supplementary information

Supplementary Information

Reporting Summary
