# Supplementary material for: Reply to: Assessing the precision of morphogen gradients in neural tube development
Source: Nat Commun. 2024 Feb 1;15:930. doi: 10.1038/s41467-024-45149-7 (PMC10834396; doi:10.1038/s41467-024-45149-7)
Supplement: Supplementary file 1 — Supplementary Information [file 41467_2024_45149_MOESM1_ESM.pdf]

Supplementary Information for:
Reply to:
Assessing the precision of morphogen gradients in neural tube development

Roman Vetter[1,2] and Dagmar Iber[1,2]

[1]Department of Biosystems Science and Engineering, ETH Zürich, Mattenstrasse 26, 4058 Basel, Switzerland
[2]Swiss Institute of Bioinformatics, Mattenstrasse 26, 4058 Basel, Switzerland

In this supplementary document, we respond to the criticism by Zagorski et al. on our article [1] point by point. The Matters Arising manuscript by the authors is reprinted in bold face, and our response follows each point in Roman.

In a recent publication, Vetter and Iber present theoretical analysis of morphogen gradient precision in the vertebrate neural tube[1]. Their study is based on reanalysis of data published by Zagorski et al[2]. Here we discuss assumptions that limit these conclusions, and address inaccuracies in the analysis.

We disagree with the authors that there are inaccuracies in our analysis, as detailed below. On the contrary, our work highlights several inaccuracies in the authors' 2017 Science paper [2].

In multiple systems, morphogen gradients have been studied by measuring fluorescent reporters of signaling activity in fixed tissues[3]. A common practice is to estimate the imprecision of a gradient by assessing the variation in fluorescent intensity (FI) between individual embryos at every position in the tissue[4]. The positional error σ_x of the gradient is approximated by multiplying the variation of morphogen levels σ_C by the local gradient steepness $\left|\frac{\partial C}{\partial x}\right|^{-1}$ at that position: $\sigma_x \approx \left|\frac{\partial C}{\partial x}\right|^{-1} \sigma_C$. Vetter and Iber point out that different methods for estimating the local gradient steepness can produce different results. One method, numEPM, uses the spatial derivative of mean intensity at the position of interest. Another method, fitEPM, assumes that the mean gradient is exponential. In this case, the local steepness of the gradient is given by the fitted mean intensity at a position divided by fitted exponential decay length. A third method, DEEM, estimates the positional error as the standard deviation of positions $x_{\theta,i}$ that correspond to a defined concentration threshold: $\sigma_x = SD\{x_{\theta,i}\}$. The DEEM method is derived from the mathematical definition of positional error and hence considered to represent the most direct measure of positional error from an ensemble of gradients.

For low FI values, close to background levels, numEPM and fitEPM methods are influenced by how background FI is estimated and subtracted and by how data is binned

and smoothed along the positional axis. Thus, in the tail of a gradient, the positional error estimates generated by the two methods are inexact and may differ.

Firstly, challenges of background subtraction and smoothening apply also to DEEM. As the authors confirmed (A. Kicheva, personal communication, Supplementary Information), they employed 8-bit imaging, so that the flat tail of their gradients reflects the detection limit of their chosen imaging depth. Under such circumstances, any computational analysis is meaningless beyond the detection limit.

Secondly, the differences between the computational methods exist independently of any measurement consideration. FitEPM yields the wrong positional error whenever the mean of the gradients deviates from an exponential function. The gradients that have been reported by Zagorski et al. are fitted well by exponential functions close to the source, and are roughly constant in the center of the domain. In both cases, FitEPM yields the wrong result further away from the source as neither the mean of exponential functions nor the mean of constant functions is an exponential function.

Vetter and Iber claim they can determine which of the two methods is correct by testing which method gives the result closest to estimating the precision of an artificial dataset consisting of an ensemble of exponential gradients using the DEEM method. Comparing the positional error of this simulated dataset to the two methods, they conclude that NumEPM is correct while FitEPM overestimates the positional error. This conclusion depends on the assumption that experimental gradients are perfectly exponential.

It is an indispensable pillar of quantitative science to test the accuracy of methods by applying them to problems for which the result is known. If a method fails to provide the correct results for known problems, it is unreasonable to apply it to similar problems, for which the correct answer is not known. In this spirit, we are comparing the three methods (FitEPM, NumEPM, DEEM) using a set of exponential functions, as for those, the result is known. We are *not* claiming that morphogen gradients are necessarily perfectly exponential. What we are showing is that for exponential functions, FitEPM fails to yield accurate results (except close to the source), and thus it cannot be expected to work for similarly shaped noisy gradients.

To compare the positional errors of morphogen gradients and of their readout, the same method must be used to calculate both of them. The authors chose DEEM to quantify the positional error of the readout. DEEM is unrelated to the shape of the gradients or their readouts. Unlike DEEM, the two other methods, NumEPM and FitEPM, estimate the standard deviation of the position indirectly, via error propagation. We can therefore evaluate whether NumEPM and FitEPM are appropriate by comparing their results to DEEM, when applied to data. As we show, NumEPM gives very similar results as DEEM, while FitEPM greatly overestimates the positional error for sets of exponential gradients that have the same variability in their two parameters (amplitude and gradient length) as reported by Zagorski et al., based on their own fitting of their gradient data with exponential gradients. In fact, the positional error that we obtain with FitEPM

for the perfectly exponential gradients is very similar to the results reported by Zagorski et al. based on the measured, noisy data (Fig. 1D).

Further away from the source, the reported gradient shapes lose their exponential shape and become flat. Here, FitEPM performs even worse.

The cellular response to the signal and tissue heterogeneities result in gradient shapes that deviate from a perfectly exponential curve[5,6].

We agree with this statement, and for non-exponential gradients, the mean gradient will potentially deviate even further from an exponential function, introducing an even greater error when using FitEPM.

The poor signal-to-noise ratio in the gradient tail means that the real shape of gradients in this region cannot be reliably measured.

It is not just that the gradient cannot be reliably measured, but rather not be detected at all, except close to the source. Given the author's use of 8-bit imaging, which limits the detectable signal range to 256-fold, the reported flat gradient "signal" is likely just technical noise.

The fact that the measured gradients turn from an exponential shape to a flat noisy shape near the 8-bit limit led us to suspect that the reported poor signal-to-noise ratio is most likely the consequence of an inappropriate imaging regime. The imaging depth is not provided in the methods section of [2], and was inaccessible to us prior to the publication of our article. The authors meanwhile confirmed that they had used 8-bit imaging (A. Kicheva, personal communication, Supplementary Information). It is unclear to us why the authors nonetheless insist that after 30 hours, morphogen gradients are too imprecise to inform patterning in the center of the neural tube — even though the employed 8-bit imaging depth does not allow to image the gradient.

Thus, judging the "correctness" of the two methods by comparison to an artificial dataset, which may not represent the true shape of gradients in the tissue, is likely to be misleading.

As we explained above, the same mathematical analysis method needs to be used when evaluating the positional error of gradient and readout. This was not done by Zagorski et al., but a method was used that even with perfectly exponential gradients wrongly yields huge positional errors unless the gradients are identical. Our analysis shows that when the same methods are used for both the gradient and readout, their positional errors are compatible with each other.

More importantly, the analysis of Vetter and Iber indicate that there is in fact very good agreement between the precision estimated by the different methods during the relevant stages of neural tube development (0-15ss, corresponding to 0-30h). An examination of the data (Fig 1E in Vetter and Iber) shows that the two methods produce identical

precision estimates for time points 0-5ss. For 10-15ss, the estimates are also very similar and diverge only in the gradient tail: where DEEM and numEPM estimate 5-6 cell diameters, fitEPM results in 6-8 cell diameters. These positional errors occur at distances greater than 60% tissue length from the morphogen source for GBS-GFP and 45% for pSmad.

As we pointed out in our article, it lies in the nature of the method that the mean of exponential gradients is fitted well by an exponential function close to the source, but not further away. Our paper does not claim that FitEPM is inaccurate early on or very near the source, but later on and away from the source.

Zagorski et al. were not able to image the exponential gradients beyond 15 SS because they employed 8-bit imaging, which allowed them to detect fluorescence intensity changes only over a 256-fold range. Beyond the 8-bit limit (~ 110 μm), their imaging returns technical noise, likely explaining the sharp transition from an exponential to a flat gradient shape. Given this technical detection limit, it is impossible to know whether the reported flat part of the gradient reflects biological reality or the technical limits of 8-bit imaging (although the latter seems more likely to us). No matter which of the two scenarios applies, FitEPM will return a wrong positional error in the flat part of the reported gradients. FitEPM relies on an exponential fit to the mean signal, and this fit is dominated by the higher signal close to the source. As such, FitEPM neither provides a correct estimate of the positional error if the gradients were indeed flat, nor for the case that the exponential gradients continued with the same gradient shape as near the source (but cannot be detected by 8-bit imaging). We analyzed the latter scenario via error propagation methods in our article and showed that the expected positional error of the gradients would be largely consistent with that of the readouts if the gradients indeed remained exponential.

In summary, by employing 8-bit imaging and using FitEPM over the entire spatial and temporal range for the gradients, but DEEM for the readouts, the 2017 Science paper wrongly concludes that single gradients become too imprecise to pattern the center of the neural tube.

This similarity in the estimates at early stages is relevant, because, as we show in Zagorski et al[2], early (before 15 ss) but not late stage gradients are used to establish pattern.

The authors provide no such evidence in their paper. In fact, in a previous paper [3], Kicheva and Briscoe show that late interference with SHH signaling still impacts OLIG2 levels, in agreement with previous work by others [4].

In Zagorski et al[2], we derive a decoding map of Shh and BMP signaling using the profiles measured at 5ss and validate key properties of the map with independent experiments.

The part of our paper that addresses the authors' 2017 Science paper is only concerned with the correct method to compare the positional errors of gradients and their readouts, not with the decoding map. But as the authors bring this up, we emphasize that the decoding map would not

work to explain the position of domain boundaries were the authors to use their measured gradients at time points other than the very first. At these later time points, at least one of the two gradient profiles reported by Zagorski et al. is flat in the part of the domain where the NKX6.1 and PAX3 boundaries are observed. Consequently, their reported gradients could not define the readout position, whether they use a decoding map based on opposing gradients or a direct threshold-based readout of a single gradient.

We demonstrate that the downstream transcriptional network requires morphogen input for less than 30h to generate the pattern and this mechanism is sufficient to maintain gene expression in the absence of ongoing signaling at late stages.

Despite all their quantitative work, the authors have never shown that this transcriptional network yields the correct progenitor domain boundaries when the measured gradients are used as input. Even though Zagorski et al. claimed above that our use of exponential gradients was unrealistic, they used an idealized exponential gradient, rather than their measured gradients, as input, and did not quantitatively match their boundary data over time. But again, this is of no concern regarding the topic of our analysis, which focuses on a consistent way to compare the positional errors of gradients and their readouts.

This reinforces previous experimental evidence indicating that the temporal window for morphogen-dependent cell fate specification is during the first 30h of mouse neural tube development[7]. Thus, for the time interval that is relevant for pattern formation, the fitEPM, NumEPM and DEEM methods produce similar estimates of positional error.

The conclusion in the cited publication [3] hinges on a mathematical analysis that is inaccessible to us.

The claim that the gradients were too imprecise beyond the first 30h hinges on the use of a mathematical flawed method, and on 8-bit imaging that makes it impossible to detect exponential gradients beyond this point, as detailed above.

Vetter and Iber also argue that imprecision of the signaling gradients is overestimated by grouping signaling profiles into temporal bins that correspond to 10h of developmental time. For a given bin, all signaling profiles are assumed to have the same DV length. Vetter and Iber suggest that this introduces a “scaling error”. To define it, they assume that all profiles in the temporal bin have equal amplitudes and decay lengths, but different absolute lengths. They reason that any resulting positional error is therefore the product of the differing lengths, rather than actual variability in the amplitude and decay length.

This is a misinterpretation of our work. In the quantitative part (Fig. 2C-E,H-J), we are using the variability in the amplitude and gradient length that the authors reported in their 2017 Science paper. Only to illustrate the concept of the scaling error in the schematics of Fig. 2F,G, the same C_0 and λ are used for visual clarity. This variability was determined *after* the gradients had been

scaled. We are correcting for this effect by exploiting the reported uniform NT growth behavior. But we agree that this correction can, unfortunately, only be approximate.

This reasoning is problematic in several ways. First, if the signaling gradient profiles are corrected in this way, then the gene expression boundaries of Pax3 and Nkx6.1 also need to be corrected, as both genes have graded expression profiles before 15ss[2]. This is something that Vetter and Iber did not do. Instead they compare the corrected signaling gradients to the imprecision of Pax3 and Nkx6.1 as reported in Zagorski et al., that is without correction.

The information whether Zagorski et al. also scaled the NT length in each 5-somite bin before evaluating the PAX3 and NKX6.1 boundaries, and whether they would therefore need to be corrected as well, was inaccessible to us before publication of our paper. When we determined the positional error of the PAX3 and NKX6.1 domain boundaries ourselves using our own imaging data, we could reproduce the reported positional error only when we binned, but *did not* scale the domains. Having said this, this could, of course, be the result of differences between mouse lines. Based on this finding and the different methods that Zagorski et al. used to calculate the positional error for gradients and readouts, we concluded that it is most plausible that they measured the PAX3 and NKX6.1 boundaries on unscaled domains.

During the writing of our response to their Matters Arising letter, the authors informed us that they did not scale the readout data (A. Kicheva, personal communication, Supplementary Information). The raw data and scripts that would allow us to check this remain inaccessible to us.

The remaining difference is in any case small (at most one cell diameter), and we expect that additional effects from the epithelial structure (pseudostratification) amplify the measured positional error [5], and that, when carefully remeasured, the positional errors of both gradients and readouts will turn out to still be lower. As we show in follow-up work, the expected positional error of the morphogen gradients is even lower when considering that they are not spreading on a 1D domain, but at least in 2D [6].

Furthermore, by subtracting the scaling error, Vetter and Iber assume that it has an additive contribution to the overall profile variability. This excludes the possibility that variability in decay length and amplitude could dominate any scaling variability. In such a scenario, subtracting the scaling error would lead to unrealistic underestimation of the actual error. Taken together, the proposed "scaling error" correction is applied inconsistently and might underestimate the actual variability.

Given our lack of access to the authors' raw imaging data, the degree of additivity of the scaling error in the authors' methodology on the biological positional error remains unknown to us. However, as we show in our article, the positional error of the gradients and the readout are comparable even if the errors are only partially additive.

The arguments that Vetter and Iber develop lead them to conclude that gene expression boundaries in the neural tube are positioned by a single morphogen gradient, rather than the combined interpretation of both signaling pathways.

Our work shows that single gradients *can* provide sufficient positional information in the neural tube. We do not claim that other precision-enhancing effects are excluded, nor that gene expression boundaries are positioned by just one of the morphogen gradients in the neural tube.

Implicit in this idea is that cells somehow distinguish which of two independent gradients is the most precise and use that to determine their identity.

We do not make such an assumption. It appears to us that the authors might have misinterpreted our Fig. 6A,D,E to arrive at this impression. In Fig. 6B,D,E we explicitly show that such an assumption is not necessary.

This misses a crucial point. There is direct experimental evidence that neural progenitors respond to combinations of signaling factors. In Zagorski et al[2], we show that neural progenitor cells adopt molecular identities that depend on the levels of both BMP and Shh signaling. This is a key observation that motivates our analysis of how neural progenitors use opposing gradients to decode their position.

We do not challenge these experimental observations that actually go back to [7]. However, there is no evidence that they serve to increase precision. In fact, as shown by others [8], also the ventral-most NKX2.2 domain responds to BMP, even though according to the analysis by Zagorski et al., the BMP gradient would be way too noisy to yield any useful positional information there.

Vetter and Iber go on to suggest that gradient variability can be accurately inferred from “summary statistics of exponential gradients”. This assumes that gradients are strictly exponential. However, diffusion and degradation often depend on the expression of molecules that are regulated by morphogen signaling itself. This type of feedback can result in deviations from exponential gradient shape[8].

In this second part of our paper, we developed an error propagation formula to estimate the positional error far from the source (where measurements have so far reached technical limits) based on the variability that can be measured near the source. Whether or not gradients are exponential is indeed an open question. For those cases, in which gradients do remain exponential, our formula can be used. Recognising that they may not necessarily need to do so in other systems of gradient-based patterning, we also presented a simulation framework in the final part of our paper that can be used to estimate the positional error also for more complex cases, as the authors refer to. Using this simulation framework, we have recently shown that non-linear decay, which results in non-exponential gradients, and which had previously been proposed to increase the robustness to variability in the source [9], yields very similar results as linear decay, and does not result in a relevant increase in precision [10]. Spreading in 2D rather

than in 1D, however, substantially reduces gradient variability and therefore permits subcellular precision for physiological levels of molecular noise [6]. Together, this reinforces our previous findings that single morphogen gradients can be sufficiently precise to pattern the mouse neural tube, also when considering additional physiological aspects.

Furthermore, in their analysis, Vetter and Iber assume that ligand and signaling gradients have comparable variability and that any discrepancy results from technical measurement errors. This ignores the possibility that the signal transduction mechanisms alter the noise properties of a signal[9].

We do not make such an assumption. However, if the readout was more precise than the gradients, then the missing information would have to be introduced somehow. Zagorski et al. proposed that this missing information is obtained by reading out both gradients simultaneously in the center of the domain. As we show, single gradients can be sufficiently precise to define the progenitor domain boundaries also in the center of the NT, offering a simple explanation of how patterning is controlled in the NT.

Zagorski et al. themselves make the assumption that the GBS-GFP and pSMAD gradients can serve as proxies for the SHH and BMP gradients, even though GBS-GFP may respond very differently from more dorsal SHH-dependent genes, as we explained in detail in our paper.

The statistical arguments that Vetter and Iber use also rely on the assumption that all variables are independent and uncorrelated. For instance, C0 and lambda are assumed to vary independently. Given that both C0 and lambda depend on D and k, this assumption is incorrect. Indeed assessing the correlation between D and k for measurements taken from 5- 25ss embryos reveals a modest but significant correlation of R=0.26 (p=0.001). This is inconsistent with the assumption C0 and lambda vary independently.

It is unclear from where the authors take the information regarding a correlation between D and k, as no reference is given, and thus, on what published report of a correlation we could have based our statistical analysis on. What we do observe is that there is no significant correlation in the published measurements of C_0 and λ [11]. As the data was plotted using vector graphics, we could extract the plotted point pairs, from which we determined Pearson's $R=-0.0061$ ($p=0.94$) and Kendall's $\tau=0.056$ ($p=0.26$) as reported in our paper, suggesting that any correlation between C_0 and λ, if it exists, is negligible. This is confirmed by our cell-based simulations (Fig. 1): The values of C_0 and λ, that we determined from fitting the numerically simulated gradients with independent noise in the three kinetic parameters, are largely uncorrelated: $R=-0.082$ ($p=0.010$) and $\tau=-0.053$ ($p=0.012$). This confirms the validity of our assumption.

In conclusion, the assumptions inherent to the work of Vetter and Iber and their decision not to take into account key experimental evidence make their conclusion, that gene expression boundaries in the neural tube are accurately positioned by a single morphogen gradient, unconvincing.

None of the points of criticism raised by Zagorski et al. bears relevance to the conclusions of our article, and as pointed out above, we clearly state that our work shows that single gradients *can*, in principle, be precise enough to encode patterns in the neural tube. We will discuss *how* the progenitor domains are defined in forthcoming work.

In summary, we demonstrated that the conclusion of Zagorski et al., that the positional error of gradients is much higher than that of their readouts, is the consequence of using different methods to calculate them, and of using 8-bit imaging that can detect fluorescent signals only over a 256-fold range. When using consistent methods, the positional errors are very similar within the distance from the source where 8-bit imaging can yield technically sound results.

Supplementary References

1. Vetter, R. & Iber, D. Precision of morphogen gradients in neural tube development. *Nat. Commun.* **13**, 1145 (2022). DOI: 10.1038/s41467-022-28834-3
2. Zagorski, M. et al. Decoding of position in the developing neural tube from antiparallel morphogen gradients. *Science* **356**, 1379–1383 (2017). DOI: 10.1126/science.aam5887
3. Kicheva, A. et al. Coordination of progenitor specification and growth in mouse and chick spinal cord. *Science* **345**, 1254927 (2014). DOI: 10.1126/science.1254927
4. Yu, K., McGlynn, S. & Matise, M. P. Floor plate-derived sonic hedgehog regulates glial and ependymal cell fates in the developing spinal cord. *Development* **140**, 1594–1604 (2013). DOI: 10.1242/dev.090845
5. Iber, D. & Vetter, R. Relationship between epithelial organization and morphogen interpretation. *Curr. Opin. Genet. Dev.* **75**, 101916 (2022). DOI: 10.1016/j.gde.2022.101916
6. Long, Y., Vetter, R. & Iber, D. 2D effects enhance precision of gradient-based tissue patterning. *iScience* **26**, 107880 (2023). DOI: 10.1016/j.isci.2023.107880
7. Liem, K. F., Jessell, T. M. & Briscoe, J. Regulation of the neural patterning activity of sonic hedgehog by secreted BMP inhibitors expressed by notochord and somites. *Development* **127**, 4855–4866 (2000). DOI: 10.1242/dev.127.22.4855
8. Mizutani, C. M., Meyer, N., Roelink, H. & Bier, E. Threshold-dependent BMP-mediated repression: A model for a conserved mechanism that patterns the neuroectoderm. *PLoS Biol.* **4**, e313 (2006). DOI: 10.1371/journal.pbio.0040313
9. Eldar, A., Rosin, D., Shilo, B.-Z. & Barkai, N. Self-enhanced ligand degradation underlies robustness of morphogen gradients. *Dev. Cell* **5**, 635–646 (2003). DOI: 10.1016/S1534-5807(03)00292-2
10. Adelmann, J. A., Vetter, R. & Iber, D. Patterning precision under non-linear morphogen decay and molecular noise. *eLife* **12**, e84757 (2023). DOI: 10.7554/eLife.84757
11. Cohen, M. et al. Ptch1 and Gli regulate Shh signalling dynamics via multiple mechanisms. *Nat. Commun.* **6**, 6709 (2015). DOI: 10.1038/ncomms7709
